# Differential metabolic alterations in IDH1 mutant vs. wildtype glioma cells promote epileptogenesis through distinctive mechanisms

**DOI:** 10.3389/fncel.2023.1288918

**Published:** 2023-11-09

**Authors:** Darrian McAfee, Mitchell Moyer, Jaden Queen, Armin Mortazavi, Ujwal Boddeti, Muzna Bachani, Kareem Zaghloul, Alexander Ksendzovsky

**Affiliations:** ^1^Department of Neurosurgery, University of Maryland School of Medicine, Baltimore, MD, United States; ^2^The College of Arts and Sciences, Cornell University, Ithaca, NY, United States; ^3^Department of Neurosurgery, University of Michigan, Ann Arbor, MI, United States; ^4^Surgical Neurology Branch, National Institute of Neurological Disorders and Stroke (NINDS), National Institutes of Health, Bethesda, MD, United States

**Keywords:** tumor-related epilepsy, brain, glioma, seizure development, tumoral metabolism, peritumoral excitability

## Abstract

Glioma-related epilepsy (GRE) is a hallmark clinical presentation of gliomas with significant impacts on patient quality of life. The current standard of care for seizure management is comprised of anti-seizure medications (ASMs) and surgical resection. Seizures in glioma patients are often drug-resistant and can often recur after surgery despite total tumor resection. Therefore, current research is focused on the pro-epileptic pathological changes occurring in tumor cells and the peritumoral environment. One important contribution to seizures in GRE patients is metabolic reprogramming in tumor and surrounding cells. This is most evident by the significantly heightened seizure rate in patients with isocitrate dehydrogenase mutated (IDH^mut^) tumors compared to patients with IDH wildtype (IDH^wt^) gliomas. To gain further insight into glioma metabolism in epileptogenesis, this review compares the metabolic changes inherent to IDH^mut^ vs. IDH^wt^ tumors and describes the pro-epileptic effects these changes have on both the tumor cells and the peritumoral environment. Understanding alterations in glioma metabolism can help to uncover novel therapeutic interventions for seizure management in GRE patients.

## 1. Introduction

Approximately 90% of patients with low-grade gliomas (LGG) and 50% with high-grade gliomas (HGG) experience glioma-related epilepsy (GRE), defined by the occurrence of spontaneous seizures with clear glioma correlation (Prakash et al., 2012; You et al., 2020). The presence of seizures in a glioma patient is a positive predictor of reduced mortality (Vecht et al., 2014; Ge et al., 2022). However, worsening seizures, specifically seizure recurrence following a period of seizure freedom or increased seizure frequency, is a positive predictor of glioma progression in patients with established GRE (Smits and Duffau, 2011; Prakash et al., 2012). This is supported by recent findings suggesting that gliomas, and other brain tumors, promote epileptogenesis in the surrounding peritumoral brain tissue through structural alterations of existing neural connections and through molecules released by tumors (Adhikari, 2021; Komiyama et al., 2022). Furthermore, recent observations from intracranial electrocorticography (ECoG) studies in glioma patients have shown that seizure onset zones (SOZs) are often located 1.5 cm beyond the tumor margin (Mittal et al., 2016). Thus, understanding the impact of gliomas on the peritumoral environment will allow us to better elucidate the pathophysiology of GRE and inform targeted therapeutics.

The peritumoral neuronal hyperexcitability associated with gliomas has been attributed to a large number of factors such as mass effect, disruption of ion homeostasis, blood brain barrier breakdown, neuroinflammation, genetic mutations, and metabolic alterations (Hills et al., 2022; Goethe et al., 2023; Tobochnik et al., 2023). One major mechanism through which tumors promote epileptogenicity is by metabolic reprogramming in tumor cells and in the peritumoral environment. Glioma cells require a significant energy supply to sustain a higher metabolic rate (Bi et al., 2020). In response to this increased energy demand, tumor cells reprogram their carbohydrate metabolism to primarily utilize glycolysis in lieu of oxidative phosphorylation, regardless of tissue oxygenation. This phenomenon is known as aerobic glycolysis, or the Warburg effect, and results in more rapid adenosine triphosphate (ATP) production. In addition to providing an energy source, metabolic changes in tumoral cells can impact the peritumoral environment through the release of downstream metabolic byproducts, as well as through other alterations such as relative peritumoral hypoxia (You et al., 2012; Mortazavi et al., 2022; Giambra et al., 2023).

**Figure 1:**
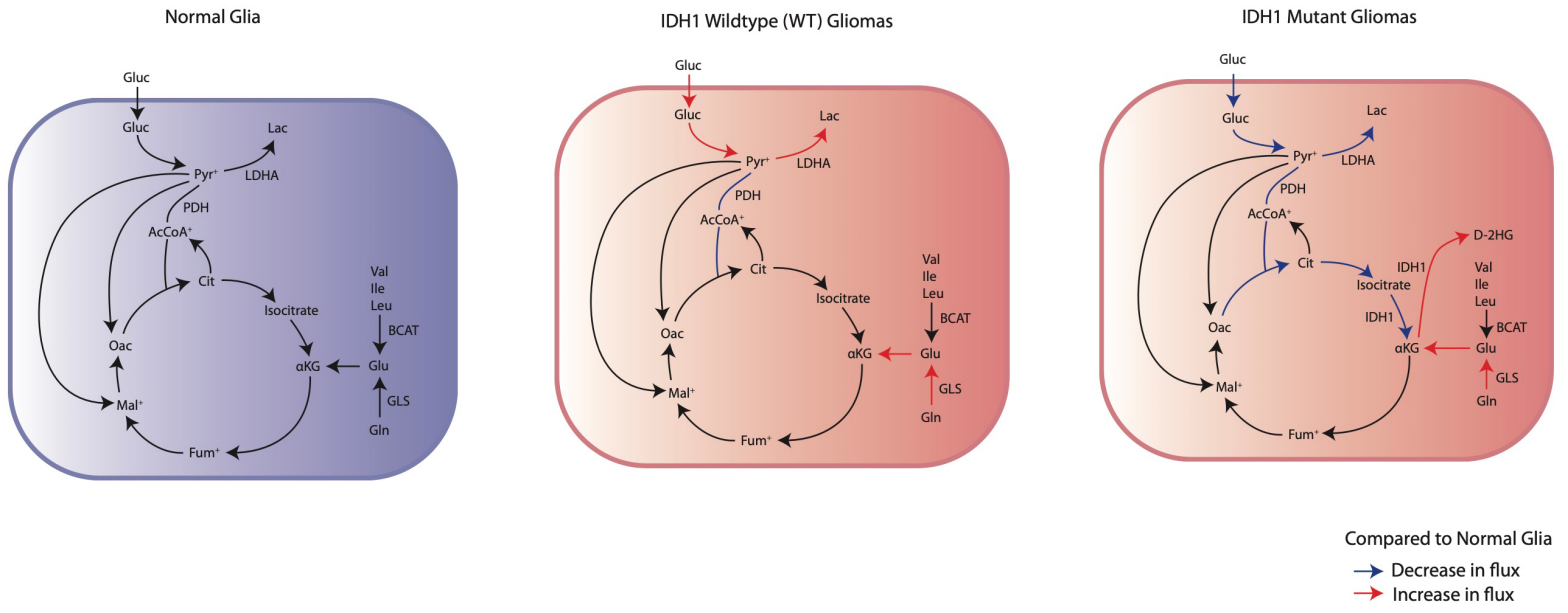
Comparison of the changes in IDH^wt^ glioma cells and IDH1^mut^ glioma cells energy metabolism to normal glia. IDH^wt^ glioma cells have an upregulation of aerobic glycolysis through increased glucose consumption and LDHA expression. IDH1^mut^ glioma cells demonstrate suppression of glycolysis and subsequent reliance on oxidative phosphorylation through glutaminolysis for anaplerosis.

In the context of gliomas, metabolic reprogramming in tumor and peritumoral cells has significant clinical implications in patient symptomology and prognosis. One example of this is the identification of mutations in isocitrate dehydrogenase 1 (IDH1) and/or 2 (IDH2) as critical biomarkers for glioma classification and prognosis (Weller et al., 2015). Interestingly, IDH^mut^ glioma patients having a preoperative seizure rate of 59-74%, compared to 18-34% seen in IDH^wt^ glioma patients (Chen et al., 2017). The higher frequency of seizures in IDH^mut^ tumors suggests that understanding the metabolic differences between IDH^wt^ and IDH^mut^ glioma cells could reveal new mechanisms causing epilepsy and lead to targeted advancements in GRE detection and seizure management. This review will focus specifically on the metabolic reprogramming that occurs in tumoral cells based on IDH mutation status (Figure 1) and describe the corresponding pro-epileptic effects on the surrounding brain tissue.

## 2. Pro-seizure metabolic reprogramming in isocitrate dehydrogenase wildtype glioma

Increased energy needs in glioma cells contribute to epileptogenicity by altering the metabolism of both tumor and surrounding cells. Stabilization of hypoxia inducible factor 1α (HIF-1α) is a primary metabolic response in IDH^wt^ cells (Strickland and Stoll, 2017). While hypoxia usually activates HIF-1α, in gliomas, HIF-1α stabilizes even before hypoxia occurs in the tumor or surrounding cells, indicating its independence from hypoxia (Woolf and Scheck, 2012; Strickland and Stoll, 2017). This stabilization of HIF-1α fosters metabolic adjustments, including higher glucose uptake and increased aerobic glycolysis, while reducing oxidative phosphorylation (Rempel et al., 1996).

**Figure 2:**
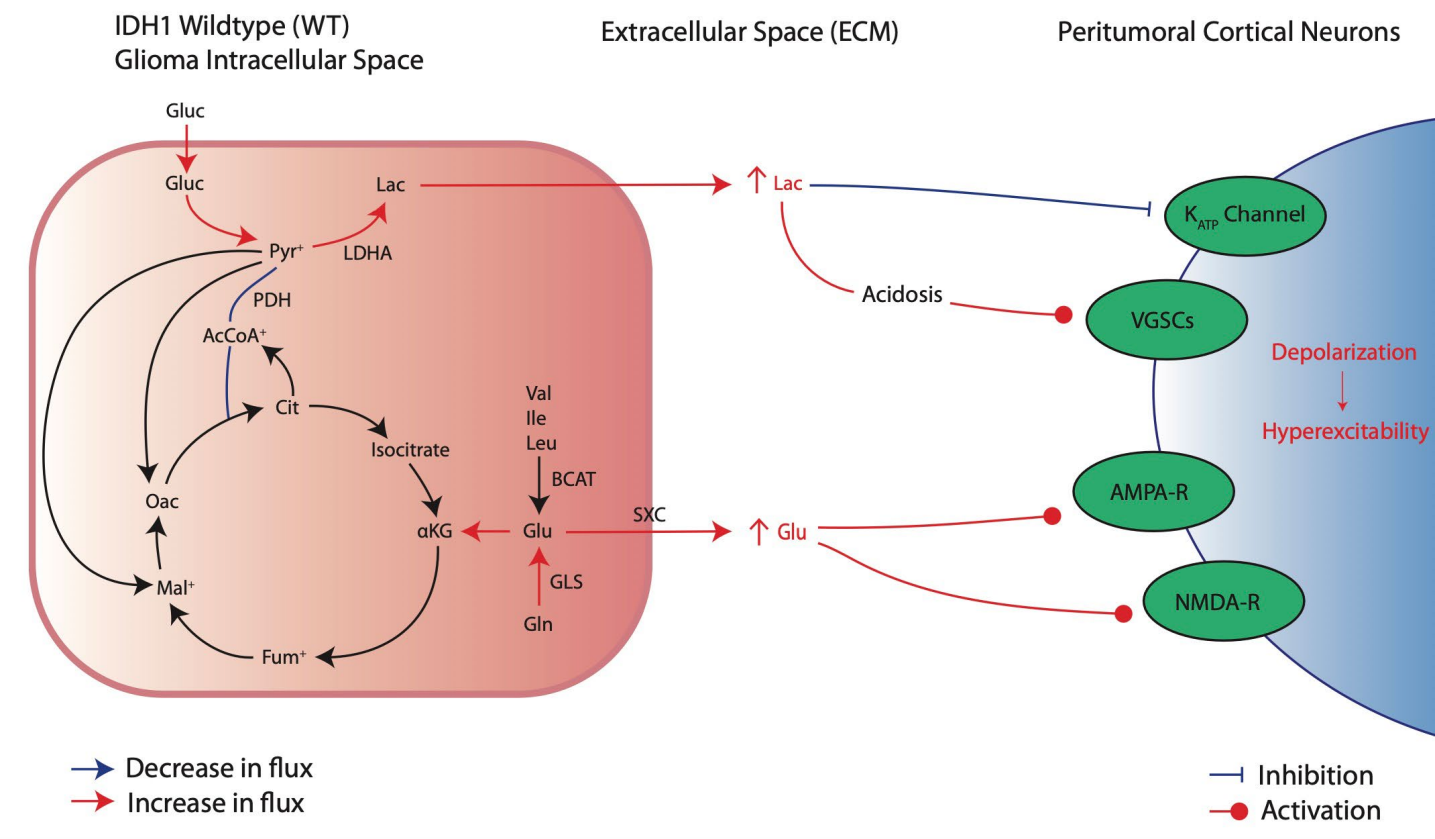
Metabolic reprogramming in IDH^wt^ glioma cells and their pro-convulsive effects. IDH^wt^ cells upregulate aerobic glycolysis and increase extracellular lactate through increased LDHA activity, leading to local acidosis and peritumoral neuronal depolarization of local cortical neurons. In addition, increased glutamate production and release induces local activation of excitatory receptors AMPA and NMDA.

In normal glucose metabolism, glucose enters neurons mainly through facilitated diffusion glucose transporters (GLUTs) and is converted to pyruvate through a series of glycolytic steps (Yu et al., 2016). Pyruvate is then preferentially converted to acetyl-CoA by pyruvate dehydrogenase (PDH) which enters the tricarboxylic acid (TCA) cycle to generate electron carriers NADH and FADH_2_ for ATP production by the electron transport chain through oxidative phosphorylation (Yu et al., 2016). Normally, in anaerobic conditions, pyruvate can also be converted to lactate through the enzyme lactate dehydrogenase (LDH) to produce NAD^+^ needed to sustain glycolytic production of ATP (Yu et al., 2016). In glioma cells, however, HIF-1α stabilization causes pyruvate to be preferentially converted to lactate, even in aerobic conditions (Figure 2; Yu et al., 2016; Han et al., 2020). HIF-1α stabilization induces inactivation of PDH through increased phosphorylation by pyruvate dehydrogenase kinase (PDK) and upregulation of LDHA, the subunit of LDH that preferentially converts pyruvate to lactate (Kim et al., 2006, 2015; Strickland and Stoll, 2017; Han et al., 2020). This causes glioma cells to preferentially utilize glycolysis for ATP production in lieu of the TCA cycle and oxidative phosphorylation. Furthermore, HIF-1α stabilization also upregulates GLUT1 and GLUT3 transporter expression and phosphofructokinase (PFK1) expression, allowing for elevated glucose uptake, increased glycolytic capacity, and rapid production of the ATP needed to sustain the high metabolic demand of glioma cells (Han et al., 2020). In addition to the shunting of pyruvate away from the TCA cycle, the electron transport chain (ETC) required for oxidative phosphorylation is impaired in hypoxic glioma cells (Papandreou et al., 2006; Grassian et al., 2014). Dysfunction of all four ETC complexes is implicated in glioblastoma, whereas often complexes II and IV are preferentially involved in low grade gliomas (Strickland and Stoll, 2017). Similarly, these impairments are also reported in epilepsy patients with temporal lobe epilepsy (TLE), suggesting that ETC impairment may play a role in GRE (Waldbaum and Patel, 2010).

Metabolic shifts in glioma cells, such as LDHA upregulation, lead to pro-epileptic effects by releasing metabolites like lactate into the surrounding area, which induces excitatory changes in nearby neurons (Figure 2; Valvona et al., 2016). Lactate’s role in causing hyperexcitability is indicated by mechanisms also found in epilepsy (Angamo et al., 2017). In both tumoral cells and epileptic neurons, metabolic reprogramming mediated by HIF-1α leads to increased LDHA levels, boosting lactate production and release into the extracellular environment (Sada et al., 2020; Ksendzovsky et al., 2023). Furthermore, although still an area of active investigation, there is literature to suggest that LDHA inhibition may reduce seizure susceptibility (Sada et al., 2015). In the context of gliomas, increased lactate release by tumor cells may similarly contribute to seizure initiation. Although controversial in current literature, substantial evidence suggests that extracellular lactate elevations can induce peritumoral neuron hyperexcitation (Beaumont and Whittle, 2000; Schaller, 2005; Shamji et al., 2009; You et al., 2012). Specifically, the acidic environment that results from lactic acid buildup has been associated with neuronal membrane depolarization through increased voltage-gated sodium channel (VGSC) activation (Rudà et al., 2012; Monteiro et al., 2017; Stanke et al., 2021). Additionally, lactate may promote seizures through closure of ATP-sensitive potassium (K_ATP_) channels that normally act as an energy sensor to protect neurons from excessive depolarization (Karagiannis et al., 2021). Lactate is also often the initial fuel source during seizure activity; thus, excess lactate in the peritumoral environment may increase available energy and sustain increased activity in healthy peritumoral cells (Yang et al., 2013).

In addition to lactate, glutamate is another key metabolite released by glioma cells into the peritumoral environment in response to metabolic reprogramming (Figure 2). Although glycolysis becomes the main fate of glucose metabolism after metabolic reprogramming, glioma cells also increasingly rely on glutaminolysis to maintain some TCA cycle activity (Maus and Peters, 2017; Strickland and Stoll, 2017). The deamination of glutamine by glutaminase (GLS) produces glutamate, which then undergoes a series of reactions to replenish depleted TCA cycle substrates, through generation of α-ketoglutarate (αKG), and continue oxidative phosphorylation (Maus and Peters, 2017). Compared to normal astrocytes, glioma cells express increased levels of GLS and produce large amounts of glutamate (Yao et al., 2014). Additionally, glutamate can be synthesized from branched amino acids (valine, leucine, and isoleucine) by branched-chain amino acid transaminases (BCAT; Maus and Peters, 2017). Both pathways lead to increased intracellular glutamate synthesis for TCA cycle anaplerosis. Excess glutamate is released by glioma cells through system x_c_^-^ (SXC), an amino acid antiporter that exchanges intracellular glutamate for extracellular cysteine (Maus and Peters, 2017). Intracellular cysteine can be used for glutathione (GSH) production for protection against ROS (Maus and Peters, 2017). Thus, it is favorable for glioma cells to upregulate SXC activity at the expense of releasing excess glutamate generated due to metabolic reprogramming into the extracellular environment. The overall result is excessive extracellular glutamate that can lead to increased tumor proliferation (Lange et al., 2021), neuronal hyperexcitability, and seizures (Robert and Sontheimer, 2014).

Besides causing metabolic shifts in glioma cells, tumoral HIF-1α stabilization also triggers pro-convulsive alterations in the surrounding tissue. Specifically, HIF-1α-induced VEGF signaling stimulates local angiogenesis to nourish both the tumor and adjacent brain areas (Schaller, 2005; Colwell et al., 2017; Monteiro et al., 2017; Strickland and Stoll, 2017). Despite neovascularization, the tumor and surrounding tissue remain relatively hypoxic due to the new vascular supply’s disorganization and fragility (Dubois et al., 2014; Wang et al., 2021). Insufficient oxygen delivery in these regions hampers oxidative phosphorylation, leading to cellular hypoxia in tumor and surrounding cells, and further stabilizing HIF-1α (Kaur et al., 2005). Fragility in newly formed peritumoral vessels also leads to focal BBB permeability changes, which can promote seizure activity through vasogenic edema, inflammatory changes, and other alterations that affect ion balance in neurons (Dubois et al., 2014; Adhikari, 2021). Additionally, poor tissue perfusion also results in hypoxia and a further energy deficit ultimately leading to sodium-potassium pump (Na^+^-K^+^-ATPase) dysfunction (Gusarova et al., 2011; Xu and Fan, 2022). The Na-K-ATPase is the key transporter maintaining neuronal resting membrane potential; therefore, loss of proper Na^+^-K^+^-ATPase function leads to a more depolarized state in peritumoral neurons and increased probability of neurons to reach action potential threshold, and thus pathologic hyperactivity (Bazzigaluppi et al., 2017; Xu and Fan, 2022). Lastly, hypoxia in glioma cells is linked to excitatory effects in the peritumoral environment through expression of thrombospondins (TSP) (Wang et al., 2021). TSPs are a family of signaling molecules that regulate synaptogenesis and plasticity and are released by glial cells in ischemia (He et al., 2022). Similarly, TSP2 expression is significantly elevated in human glioma cells with marginal increases noted in the peritumoral environment, corresponding to hyperexcitability and increased excitatory synapse formation in peritumoral neuronal populations (Wang et al., 2021). Overall, HIF-1α stabilization in glioma cells induces hypoxic alterations in the peritumoral environment which lead to a pro-convulsive state.

## 3. Pro-seizure metabolic reprogramming in isocitrate dehydrogenase mutant glioma

**Figure 3:**
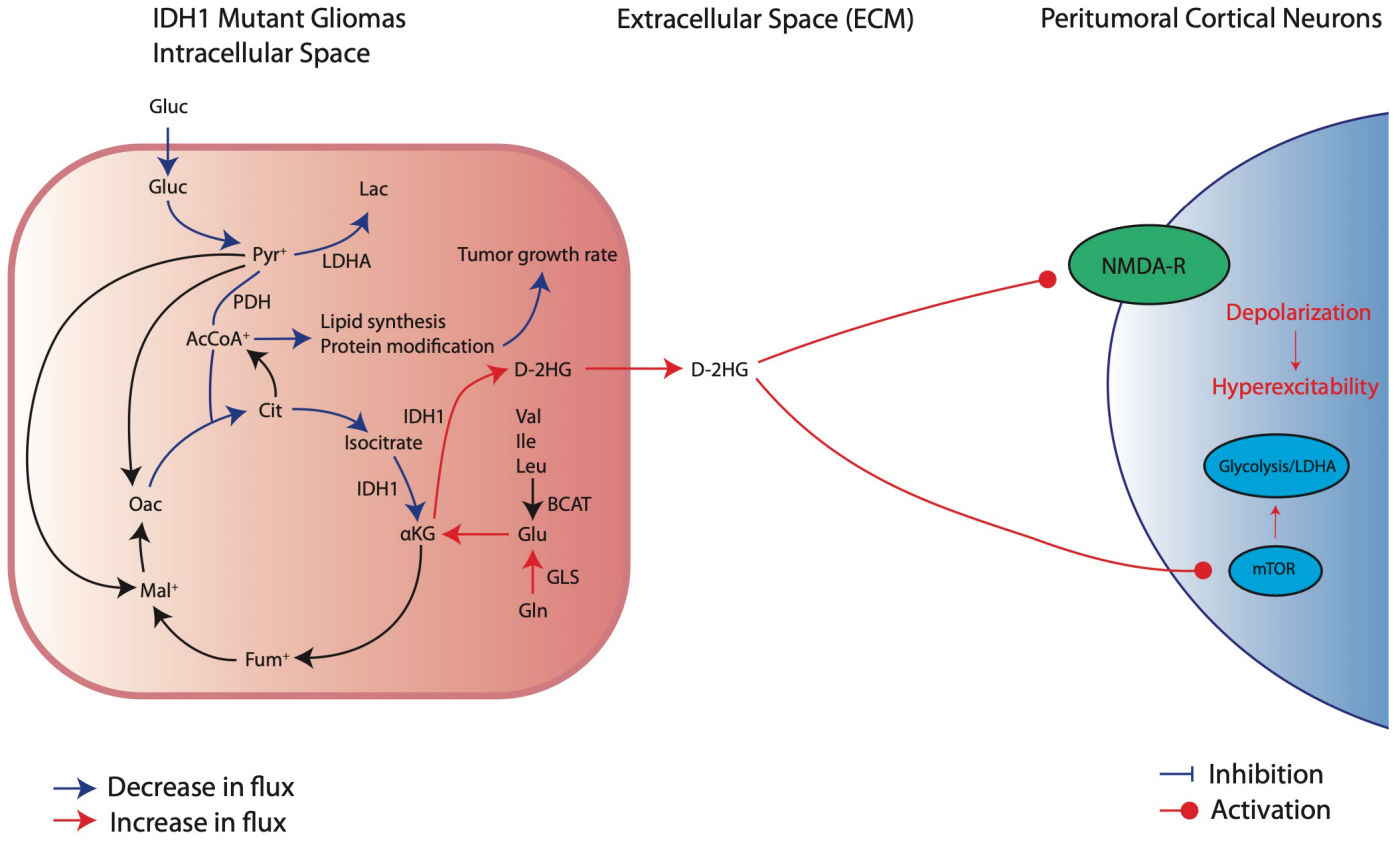
Metabolic reprogramming in IDH1^mut^ glioma cells and their pro-convulsive effects. IDH^mut^ cells have reduction in glucose consumption and downregulation of glycolytic factors, such as LDHA, which decreases carboxylic entry into the TCA cycle. Thus, IDH1^mut^ cells rely on intracellular glutamate breakdown to aKG to sustain oxidative phosphorylation. At the same time, IDH1 mutations significantly overproduce D-2HG which exerts its pro-excitatory effects through activation of NMDA receptors and the mTOR pathway of local cortical neurons.

Patients with IDH^mut^ gliomas have an increased propensity to develop GRE (preoperative seizure rate of 59-74%) compared to IDH^wt^ (18-34%) (Liubinas et al., 2014; Chen et al., 2017). In addition to the tumor-specific mechanisms described above for IDH^wt^ glioma cells, there are metabolic adaptations seen in IDH^mut^ gliomas that are distinct from those in IDH^wt^ gliomas (Figure 3). The IDH complex is usually composed of either a homodimerized complex of IDH1 subunits, in the cytoplasm, or IDH2 subunits, in the mitochondria (Han et al., 2020). In addition, there are heterotetrametric complexes that include IDH3 that also reside in the mitochondria (Yan et al., 2009). Both IDH1 and IDH2 reversibly oxidize isocitrate to αKG and reduce NADP^+^ to NADPH in the process (Dang et al., 2009; Han et al., 2020). However, mutations in either IDH1 and IDH2 decrease the binding affinity of isocitrate, increase the affinity for NADPH, and promote an irreversible neoplastic reaction that oxidizes NADPH to convert αKG to the oncometabolite D-2-Hydroxglutarate (D2HG) (Lu et al., 2012). Clinically, the vast majority (~95%) of mutations are in IDH1 rather than IDH2 (Hartmann et al., 2009). Therefore, most studies have focused on IDH1^mut^ metabolic reprogramming as opposed to IDH2. IDH1^mut^ cells can demonstrate greater than 25-fold upregulation in D2HG (Grassian et al., 2014), which can lead to many intracellular and extracellular implications in cellular metabolism and peritumoral excitability.

Unlike IDH^wt^ glioma cells, which favor mainly glycolysis with minor contribution from glutaminolysis for energy production, IDH1^mut^ cells have significantly increased reliance on glutamine breakdown to αKG for anaplerosis and maintenance of oxidative phosphorylation (Figure 3; Grassian et al., 2014). Mutations in IDH lead to silencing, rather than expression, of glycolysis related genes such as *LDHA*, *CA9*, and *VEGFA*, supporting recent findings which demonstrated a reduction in glucose consumption in IDH1^mut^ cells (Han et al., 2020; Liu et al., 2021). This silencing effect on glycolytic genes may be due to epigenetic alterations associated with D2HG accumulation or due to decreased HIF1α stabilization in IDH1^mut^ conditions, as opposed to increased HIF1α stabilization seen in IDH^wt^ cells (Zhao et al., 2009). However, there is conflicting evidence on whether HIF1α expression is increased or decreased with IDH^mut^ in glioma and more work is needed to establish the role of HIF1α in IDH^mut^ metabolic reprogramming (Han et al., 2020).

In hypoxic conditions, IDH1^mut^ cells exhibit slower growth compared to IDH^wt^ cells, which maintain their growth rate, suggesting that HIF1α responsiveness is lost in IDH1^mut^ cells (Grassian et al., 2014). This corresponds with the clinical observation that IDH mutations are present in approximately 70-80% of slower growing LGGs but only 10% of rapidly dividing glioblastomas and suggests a direct link between metabolic reprogramming and tumor growth rate (Hartmann et al., 2009; Kim et al., 2010; Turkalp et al., 2014; Louis et al., 2016). As such, IDH mutations may reduce tumor growth rate either because of reliance on a slower metabolic process in oxidative phosphorylation rather than the more rapid glycolysis, or because D2HG prevents complete reductive carboxylation of glutamine to citrate and ultimately lowers cytosolic acetyl-coA availability for cholesterol and phospholipid synthesis, amino acid modifications, and histone acetylation needed to undergo rapid cellular division (Grassian et al., 2014; Fack et al., 2017; Han et al., 2020). Some studies suggest that the slow growing nature of IDH^mut^ gliomas may lead to seizures because it induces indolent changes, rather than rapid disruptions, in peritumoral neural network connections and allows time for reorganization of tumoral and peritumoral vasculature to occur (Adhikari, 2021). Aligning with this idea, studies have found that 90% of patients with LLG experience GRE versus up to 50% of patients with HGG, and that epilepsy incidence prior to glioma diagnosis is also increased in LGG (Prakash et al., 2012; You et al., 2012; Vecht et al., 2014; Van Opijnen et al., 2023). In fact, seizures are used an independent clinical predictor of LLG, while prolonged focal neurological changes, cognitive deficits, or headaches predict HGG (Rasmussen et al., 2017). This demonstrates that metabolic adaptations associated with IDH^mut^ gliomas have direct implications on GRE development.

Although IDH1^mut^ leads to reliance on oxidative phosphorylation, these tumor cells rely predominantly on glutamine and/or glutamate rather than glucose as a primary carbon source for metabolism (Figure 3; Lange et al., 2021). Because IDH1^mut^ leads to reduced αKG for TCA function at the expense of D2HG production, these cells restore αKG by either converting glutamate directly into αKG or utilizing glutamine for αKG production indirectly by first breaking down glutamine into glutamate (Han et al., 2020). The net result of this process is that, compared to IDH^wt^ tumors, glutamate levels in IDH^mut^ tumors are substantially reduced (Han et al., 2020). This suggests that, unlike IDH^wt^ cells which may promote epileptogenesis through excess glutamate release into the peritumoral environment, IDH^mut^ cells likely do not substantially release glutamate. However, IDH^mut^ cells may promote epileptogenesis in a similar manner through release of D2HG (Figure 3).

**Figure 4:**
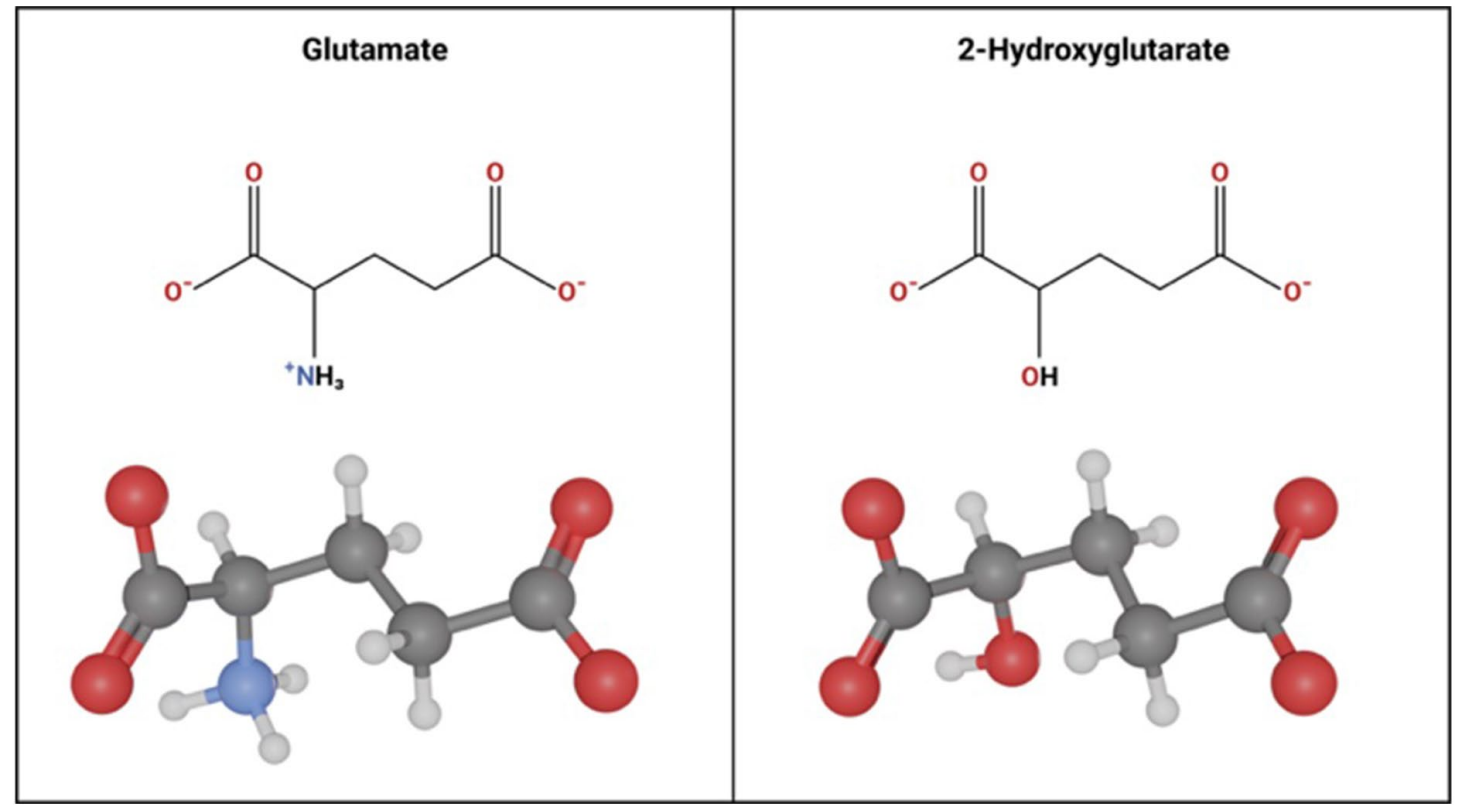
Molecular Similarity between glutamate and 2-hydroxyglutarate. Glutamate and 2 Hydroxyglutarate follow a similar molecular structure. The only difference is a hydroxyl group instead of an amine group on carbon-2 in 2-hydroxyglutarate.

Glutamate and D2HG are structurally similar molecules (Figure 4), therefore when D2HG is released into the peritumoral environment, it can bind to and activate glutamate receptors in a manner similar to glutamate (Stockhammer et al., 2012; Mortazavi et al., 2022). Activation of glutamate receptors by D2HG in turn leads to hyperexcitability and synaptic plasticity, which promote seizures and epileptic network formation (Kölker et al., 2002; Cavazos and Cross, 2006; Gong et al., 2006; Chen et al., 2017). Additionally, D2HG release by IDH^mut^ glioma cells can promote epileptogenesis independently of glutamate signaling by inducing metabolic alterations in peritumoral neurons (Mortazavi et al., 2022). Specifically, release of D2HG by tumor cells can induce aerobic glycolysis in peritumoral neurons signified by LDHA upregulation through an mTOR mediated mechanism, which in turn leads to neuronal hyperactivity and promotes seizures (Mortazavi et al., 2022). Clinically, the pro-epileptogenic effects of D2HG are supported by a related disease called 2-hydroxyglutarate-acidurea, a condition in which D2HG is elevated in the serum, cerebrospinal fluid, and urine. These patients endure persistent seizures as an initial symptom and worsen seizure burden over the disease course (Stockhammer et al., 2012; Zübarioğlu et al., 2020), suggesting that elevation of D2HG in glioma patients may contribute to GRE.

## 4. Clinical implications and future metabolism-focused innovations in GRE management

Current seizure treatment in GRE involves the use of anti-seizure medications (ASM), similar to the approach for non-tumor epilepsy patients (Chen et al., 2018). However, standard ASMs do not address the root cause and are ineffective in controlling seizures for up to 50% of GRE patients (Aaronson et al., 2011; Mittal et al., 2016). Even with complete tumor resection as an alternative when ASMs fail, 30-40% of GRE patients continue to experience seizures (Aaronson et al., 2011; Mittal et al., 2016). The continued seizures after complete tumor resection suggest either residual pathological changes in the peritumoral cortex that need post-resection treatment, or the need for improved tumor localization methods. Despite understanding the role of metabolic reprogramming in GRE-related seizure susceptibility, metabolism-specific biomarkers and therapies remain underdeveloped. We propose several approaches for clinically targeting these metabolic disruptions to identify epileptic tissue, predict outcomes, and manage seizures in GRE patients.

### 4.1. Biomarkers and treatment of peritumoral changes in IDH^mut^ GRE

Advancements in quantification of IDH^mut^ specific peritumoral changes can guide GRE prognosis and monitoring. As of now, tumor biopsy is required to determine IDH genetic status. Recently, however, less invasive measures, such as positron emission tomography (PET), have been investigated to predict IDH genotype (Lohmann et al., 2018; Liu et al., 2021). Similar research is needed to predict seizure susceptibility in glioma patients, especially IDH^mut^ glioma patients. For example, liquid chromatography–tandem mass spectrometry (MS) has been used on resected tissue to stratify patient risk for preoperative seizures based on 2HG concentration (Ohno et al., 2021). The authors identified a cutoff value of 1,190 ng/mg, above which preoperative seizures could be predicted within IDH^mut^ glioma patients. Thus, elevated 2HG values were associated with seizures. Ongoing research is exploring less invasive methods for metabolite quantification, such as cerebrospinal fluid sampling or non-invasive imaging, to assess oncogenic metabolites released by IDH^mut^ glioma cells into the peritumoral environment (Xiao et al., 2020; Balana et al., 2022). For example, MS of CSF fluid can detect and quantify 2HG levels and stratify patient’s with IDH^mut^ (Kalinina et al., 2016). Additionally, magnetic resonance imaging (MRI) and hyperpolarized MRI (hpMRI) identification of D2HG accumulation has already been used for the non-invasive classification of IDH^mut^ gliomas (Choi et al., 2012; Leather et al., 2017; Salamanca-Cardona et al., 2017; Tietze et al., 2018). The next steps involve using CSF detection to identify predictive 2HG values for predicting seizure susceptibility and employing hpMRI to non-invasively map the peritumoral cortex affected by IDH^mut^ gliomas. This would facilitate the precise removal of epileptic zones beyond the tumor margin in GRE patients. Additionally, tracking 2HG concentrations in CSF over time could help monitor treatment efficacy and adjust ASM dosing.

While recent research findings on IDH^mut^ pathology are promising, more clinical studies focused on targeting IDH^mut^ to manage seizures in GRE patients are warranted. One important case report described a patient with refractory GRE due to an IDH1^mut^ oligodendroglioma in which the IDH1 inhibitor ivosidenib was effective in reducing the patient’s seizure frequency (Vo et al., 2022). Cases like this demonstrate the potential for metabolically-targeted anti-seizure therapies in GRE. Similarly, mTOR inhibitors have been shown to reduce IDH^mut^ glioma growth rate and D2HG levels (Batsios et al., 2019). Thus, mTOR inhibitors can potentially reduce the hyperactive effects of D2HG and may reduce seizures induced by IDH^mut^ gliomas. All in all, because IDH^mut^ gliomas carry the highest rate of seizures, IDH-focused therapies for GRE patients should be further investigated.

### 4.2. Biomarkers and treatment of peritumoral changes in IDH^wt^ GRE

In IDH^wt^ glioma patients, tumor cells’ increased reliance on glycolysis results in the release of two pro-convulsive metabolites, glutamate and lactate, into the peritumoral cortex. While some studies dispute elevated glutamate levels or their link to seizures, the majority of research using Magnetic Resonance Spectroscopy (MRS) or microdialysis indicates that glutamate is significantly elevated in both glioma tumors and the surrounding cortex. One study even associates elevated glutamate in these regions with seizures (Rijpkema et al., 2003; Roslin et al., 2003; Fan et al., 2004; Marcus et al., 2010; Yuen et al., 2012). This implies that, much like D2HG in IDH^mut^ patients, glutamate could serve as a biomarker in IDH^wt^ patients. Non-invasive imaging techniques like MRS could identify epileptic zones in the surrounding tumor tissue for surgical planning. Additionally, small studies have shown that the glutamate-sensitive AMPA receptor antagonist perampanel is effective in reducing seizures in GRE patients, suggesting that neutralizing elevated extracellular glutamate may be a viable treatment for glioma-related seizures (Vecht et al., 2017; Dunn-Pirio et al., 2018; Izumoto et al., 2018; Maschio et al., 2019; Chonan et al., 2020; Coppola et al., 2020; Lange et al., 2021). Blocking the SXC transporter responsible for glutamate release from glioma cells may also be a viable therapeutic strategy, as inhibitors of SXC have been found in preclinical epilepsy studies to be anti-convulsant (Leclercq et al., 2019). However, it remains to be seen if SXC inhibition can reduce seizure susceptibility in glioma patients.

The increased reliance on glycolysis by IDH^wt^ cells, characterized by LDH upregulation, and the LDH upregulation seen in peritumoral cells around IDH^mut^ tumors, resembles that seen in neurons within non-glioma epilepsy patient tissue (Ksendzovsky et al., 2023). As such, these shared mechanisms could guide novel treatments. The LDHA inhibitor stiropentol has been shown to reduce seizures in preclinical studies and is an FDA approved treatment option for seizure management in Dravet syndrome (Sada et al., 2015; Nickels and Wirrell, 2017) Although LDH inhibition has been proven to reduce tumor growth, its anti-convulsive effects in GRE patients have not yet been explored (Guyon et al., 2022). Furthermore, metabolic imaging of lactate with 13C-hpMRI, already used for tumor localization, may also be useful for mapping epileptic zones around tumors for surgical resection (Zaccagna et al., 2022).

## 5. Conclusion

Seizures are a common and debilitating symptom in brain tumor patients, particularly those with IDH^mut^ low-grade gliomas. Despite treatment with ASM’s, many patients continue to experience seizures even after medication use and complete tumor removal. A growing body of literature has focused on the pro-convulsive effects of metabolic reprogramming in glioma cells and has begun to shed light on potential disease-specific targeted detection and treatment options for patients with GRE. Importantly, current evidence suggests that IDH^wt^ and IDH^mut^ genotypes differentially affect tumoral metabolism and consequentially have different pathological mechanisms that cause excitability in peritumoral neuronal populations. IDH^wt^ tumors upregulate glycolysis and subsequentially release excessive glutamate and lactate into the peritumoral environment, both of which can mediate hyperexcitability in the surrounding neurons. In addition to the IDH^wt^ mechanisms, IDH^mut^ tumors, on the other hand, rely further on oxidative phosphorylation driven by glutamine/glutamate metabolism, which contributes to slower growth of IDH^mut^ tumors and release of tumoral D2HG. D2HG release promotes peritumoral hyperexcitation by stimulation of glutamate receptors and induction of glycolysis in peritumoral neurons, which causes hyperactivation via lactate production similar to that seen in IDH^wt^ tumors.

Although not the only mechanism contributing to neuroexcitability, the presence of an IDH1 mutation has demonstrated significant clinical susceptibility to seizures. Given the varying underlying pathologies due to metabolic reprogramming in tumor cells, it’s crucial to tailor diagnostic and therapeutic strategies for managing seizures in patients with IDH^wt^ versus IDH^mut^ tumors. Patients with IDH^wt^ tumors may benefit more from detection of glutamate as a biomarker of epileptogenesis, whereas D2HG detection should be used in patients with IDH^mut^ tumors. Similarly, therapies targeting glutamate release (SXC inhibitors) and action on glutamate receptors (AMPA/NMDAR receptor inhibitors) should be further explored in IDH^wt^ patients, whereas IDH^mut^ may benefit more from IDH inhibitors to prevent D2HG production. In addition, LDH detection and inhibitors may be useful in both IDH^wt^ and IDH^mut^ patients. Exploring these avenues and identifying new pro-convulsive mechanisms in tumoral metabolic reprogramming will enable clinicians to more precisely classify, monitor, and treat GRE patients, leading to earlier and improved clinical decisions that will significantly enhance patient quality of life.

## Author contributions

DM: Conceptualization, Writing – original draft, Writing – review & editing. MM: Conceptualization, Writing – original draft, Writing – review & editing. JQ: Writing – original draft. AM: Conceptualization, Writing – review & editing. UB: Visualization, Writing – original draft. MB: Supervision, Writing – review & editing. KZ: Supervision, Writing – review & editing. AK: Conceptualization, Supervision, Writing – review & editing.
